# The Evolution of Holistic Processing of Faces

**DOI:** 10.3389/fpsyg.2013.00011

**Published:** 2013-01-31

**Authors:** Darren Burke, Danielle Sulikowski

**Affiliations:** ^1^School of Psychology, Faculty of Science and Information Technology, University of NewcastleOurimbah, NSW, Australia; ^2^School of Psychology, Charles Sturt UniversityBathurst, NSW, Australia

**Keywords:** face processing, evolution, comparative psychology, communication, primates

## Abstract

In this paper we examine the holistic processing of faces from an evolutionary perspective, clarifying what such an approach entails, and evaluating the extent to which the evidence currently available permits any strong conclusions. While it seems clear that the holistic processing of faces depends on mechanisms evolved to perform that task, our review of the comparative literature reveals that there is currently insufficient evidence (or sometimes insufficiently compelling evidence) to decide when in our evolutionary past such processing may have arisen. It is also difficult to assess what kinds of selection pressures may have led to evolution of such a mechanism, or even what kinds of information holistic processing may have originally evolved to extract, given that many sources of socially relevant face-based information other than identity depend on integrating information across different regions of the face – judgments of expression, behavioral intent, attractiveness, sex, age, etc. We suggest some directions for future research that would help to answer these important questions.

Holistic and/or configural processing is frequently identified as a uniquely defining characteristic of facial identity recognition, and for many proponents of this view there is a (sometimes implicit) assumption that this is the consequence of using a specialized brain region adapted, via evolution, to perform this task. Our intention in this paper is to make explicit the implications (and non-implications) of such an assumption, to review some of the extant relevant evidence, to propose some theories of how such an arrangement could have come about, and to suggest possible future research projects that might help us to address some of the many unanswered questions. Because the clearest test of evolutionary theories involve cross-species comparisons, a large part of our aim is to re-review some of the comparative literature, with emphasis on a critical analysis of the methodology used. We believe that this is important because although face processing in general has recently been reviewed across widespread species (Leopold and Rhodes, 2010), and holistic processing has been examined across the primates (Parr, 2011), these reviews were broad in scope, and so did not focus much on the *quality* of the evidence collected, which is critical when conclusions need to be drawn from very few studies with any given species, as is typical in the comparative literature.

## An Evolved Module?

There is considerable, and occasionally vigorous, debate focused on whether face-based identity recognition is achieved via a specialized face-processing module, or whether instead the apparently “special” aspects of face processing are a consequence of the fact that ontogenetically faces are a category of object that we routinely recognize at the exemplar/subordinate level, and with which we have enormous expertise (e.g., Gauthier and Tarr, 1997; Gauthier and Bukach, 2007; McKone and Robbins, 2007; McKone et al., 2007). Although it is theoretically important to determine the role played by particular kinds of experience in shaping the mechanisms underlying facial identity processing, it is not widely appreciated that the answer to this empirical question actually holds *no* implications for the evolutionary origin of our ability to recognize individuals based on their face, and whether that ability depends on a specially evolved neural module. An evolved face-processing module, that processes faces in a holistic/configural way, is actually *just* as likely to be influenced by input as it is to develop independently of specific experiences. This is particularly so, given that the mechanism *must* alter on the basis of input in order to function effectively, and that it can be virtually *guaranteed* appropriate input during normal development.

Since individual faces *must be* learned, any identity sensitive mechanism will necessarily be affected by input, and since all normally developing individuals will be exposed to a large number of faces throughout their development, an evolved mechanism that generated holistic/configural processing *only* after lengthy exposure to objects that had to be discriminated at a subordinate level (if such evidence were found) would still likely be an evolved *face*-processing mechanism, since this would be its usual phenotypic effect, and almost certainly the effect for which it was selected, evolutionarily. And so from an evolutionary perspective, the claim that the “face-sensitive” mechanism is really just a “subordinate-level expertise” mechanism is not a counter-claim at all in the absence of some putative alternative function (or side-effect of some other function) for such a mechanism. It would be like proposing that an evolved “language” mechanism is not really a language mechanism, *per se*, but an “auditory syntactic/semantic” mechanism, or perhaps more obviously, that the heart is not really evolved *to* pump blood since it is as capable of pumping any other fluid of similar viscosity.

While the importance of learning to the development and functioning of the mechanism is not a reliable way of identifying evolved adaptations (a point recently very well made by Barrett, 2012) – many evolved mechanisms *are* learning mechanism, after all – there are established ways of identifying adaptations and these can be applied to the neural mechanism that processes faces holistically/configurally. The two common methods used by behavioral scientists to test whether a mechanism has been adapted by evolution to perform a particular function, and to attempt to understand how that evolution may have occurred, are to examine the phylogenetic and/or ecological distribution of the mechanism across species, or by generating specific, unique predictions about the mechanism that reflect its function and that would be unlikely to be true of a general process mechanism.

Ultimately, a mechanism needs to be shown to have fitness consequences (to aid survival and/or reproduction) and to be affected by particular genes to be unequivocally identified as an adaptation, but for many mechanisms these criteria are difficult to meet. For example, the effect of genes on the expression of a trait is often identified using heritability estimates (the proportion of population variance in the trait attributable to genetic variation), but if a trait has reached fixation in a population then it will show no heritability because there will be no variance to attribute to genetic influences. Fixation happens when possessing a trait is universally useful, and particularly when individuals who lack the trait are at a distinct disadvantage compared to those who have it, and so selection eventually results in the whole population expressing the trait (the trait reaches fixation). Obviously, even in the absence of identifying the particular genes responsible for a trait, and in the absence of heritability estimates, universally present, clearly functional traits, like smiling or the eyebrow raise greeting, are rightly regarded as likely evolved adaptations.

Interestingly, face-recognition ability does show substantial heritability (evidence summarized by McKone and Palermo, 2010), raising a range of questions about why it should vary across the population. As McKone and Palermo point out, the fact that face-recognition ability varies independently of IQ, and shows IQ-independent heritability, *is* evidence that it depends on an independently evolved neural module, but the fact that it shows much heritability *at all* is somewhat surprising if that module evolved to aid survival, since such traits tend to reach fixation. An interesting possibility is that the residual variation might be because face-recognition ability (or at least some of it) is sexually, rather than naturally, selected (serving mate attraction/selection functions). While natural selection tends to lead to trait fixation (since variation from the “ideal” is selected against), sexual selection tends to preserve variation in traits (Arnold, 1983), since the kinds of traits that become the subject of sexual selection (those upon which mate-choice decisions or intra-sexual competition depend) are those that accurately reveal variation in underlying quality in an informative/conditional way.

In addition to the challenges presented by trait fixation in measuring the survival and/or reproductive benefits of possessing a particular mechanism (compared with not possessing it), there are also problems associated with the fact that some adaptations are vestigial (goose-bumps or the grasp reflex in humans, for example), and so will no longer have measurable fitness consequences. Given these difficulties, mechanisms are usually identified as likely adaptations if they show signs of being designed to perform a particular function and/or vary across species in a way that maps on to known phylogenies and/or ecological demands. We will examine the evidence that holistic processing of faces meets these criteria.

## Phylogenetic Distribution

While individual recognition appears to be phylogenetically widespread, at least among social species (Tibbetts and Dale, 2007), and plays an important role in a range of social interactions, like maintaining dominance hierarchies and reuniting mates and parent-offspring pairs, most of this recognition utilizes auditory or olfactory information. Few species have actually been tested for genuinely *individual* visual recognition abilities, perhaps because for many it is simply assumed based on differential treatment of group members (mates or offspring vs. rivals, or higher- vs. lower-ranking individuals, for example). Unfortunately, such behavior need not depend on actual recognition of any particular individual (Tibbetts and Dale, 2007), since there may be other visual markers of social status or relatedness that can be used to reliably guide social behavior (size or color, for example). There *is* evidence, using discrimination learning, match-to-sample, or dishabituation techniques, that cattle (Coulon et al., 2011), horses (Proops et al., 2009), jacky lizards (Van Dyk and Evans, 2007), and chickens (Ryan and Lea, 1994) can recognize individual conspecifics based only on visual information. There is also evidence for specifically *facial* recognition of dogs and humans by dogs (Racca et al., 2010), of sheep and humans by sheep (Kendrick et al., 1995, 1996), of paper wasps by paper wasps (Tibbetts, 2002), of humans by honeybees (Dyer et al., 2005), of pigeons by pigeons (Watanabe and Ito, 1991), of a range of primates by pigeons (Phelps and Roberts, 1994), and of course of both monkey and human recognition in a range of primates (reviewed by Parr, 2011), especially old-world monkeys (typically *Maccaca* species) who form the bulk of the subjects for single cell recording studies. While such findings suggest that face-based individuation might be phylogenetically widespread (Leopold and Rhodes, 2010), it is important to examine both the training and testing methodologies employed in these studies to assess the extent to which the reported ability maps on to human facial recognition. This is especially true when evaluating not just whether other species can discriminate between individuals based on their face (although this is important to know), but whether the way in which they do this is in some way *like* the way humans do it – whether it depends on holistic/configural processing.

## Phylogenetic Distribution – Basic Recognition

To our knowledge, the only species that have *only* been tested for the basic ability to use the face (or at least the head) to recognize other individuals of their own species are cattle (Coulon et al., 2011), pigeons (Watanabe and Ito, 1991), horses (Proops et al., 2009), and paper wasps (Tibbetts, 2002). Widely different techniques and stimuli are used in these studies – from reinforcing pecking at slides of unknown individuals to counting directed aggressive acts after modifying the head color pattern with paint – but each provides convincing support for the claim that these species can individuate conspecifics using visual information from their heads, the first step in face recognition as it occurs in humans. That they are species with such a wide phylogenetic spread is a reflection of testing species-particular hypotheses about individual recognition rather than a systematic attempt to examine how widespread such recognition is. In each case, the social ecology of the species and the existence of distinctive head markings (especially color patterns) was used to predict the successful use of the head as a recognition cue, but this may be a reflection of selection for the evolution of individuating cues (Tibbetts and Dale, 2007) rather than for face-processing mechanisms *per se*. It is perfectly possible that in each case the face *markings* have evolved to capitalize on visual discrimination mechanisms that were already in place (to serve other functions), without any extra evolutionary modification of these perceptual mechanisms being necessary.

## Phylogenetic Distribution – Inversion Effects

The most common test of the use of configural processing to recognize faces is to measure the effect of inverting the stimuli (rotating them 180° in the picture plane). In humans, there is a *disproportionate* inversion effect for human faces (Yin, 1969) – many stimuli are less accurately and more slowly matched or recognized when inverted, but this is especially so for faces. The most surprising report of an inversion effect on face-recognition performance is that of Dyer et al. (2005) for honeybees discriminating human faces, and an analysis of the methods used is instructive for interpreting other reports of inversion effects (or their absence) in other species. Obviously, honeybees do not have an evolved mechanism for recognizing human faces (or even other honeybees, since this is accomplished at a colony-level of categorization, chemosensorily – Breed, 1998), and Dyer et al. were explicit about the fact that if they could nevertheless recognize individuals and show an inversion effect, then that would cast doubt on claims that humans recognize each other using a specially evolved mechanism. There is room to question the logic of this claim, since the bees could do the job in a very different way (and the inversion effect is only one marker of holistic/configural processing), but there is no real need to do so because Dyer et al. did not, in fact, demonstrate even individual recognition, let alone a disproportionate inversion effect.

Bees were rewarded (with sucrose) for approaching a single exemplar of the face of one individual (CS+) and punished (with quinine) for approaching first a schematic face (CS−) and then a single exemplar of a different individual (there were actually two CS+ and two CS− faces displayed on each trial). In unrewarded “transfer” tests bees approached the previously rewarded face significantly more often (in the 80% range) than either of two previously unseen faces. This is not really a transfer test in the traditional sense (cf. Burke et al., 2001, as an example, who tested transfer to novel examples of vertical and horizontal), in that only novel CS− stimuli were included, not novel CS+ stimuli. In fact, it seems likely that the bees may well have failed even a transfer test to different exemplars of the previously rewarded and previously punished individuals’ faces, since they had only been trained with a single exemplar of each and so were free (and in a sense *encouraged*) to base the discrimination on *any* feature (like brightness, contrast, width, presence of a blemish or shadow in a particular place, *pattern* of contrast or brightness change over time as the bees moved in front of the faces, etc.) that differentiated the stimuli (a tactic that we know pigeons use whenever possible – Watanabe, 1993). Provided the novel CS− stimuli also lacked the feature (or features) each bee relied on to select the CS+, they would also be rejected without the bees being able to genuinely recognize individuals based on their face. Similarly, inverting the faces is very likely to alter the location or patterning of the feature, or features, used to perform the discrimination, and so disrupt performance – again, without the bees recognizing individuals, let alone doing so using configural information (as Dyer et al. suggest they might). The experiment as reported seems as likely to work starting with inverted faces (or indeed any other category of object) as training stimuli, and then transferring to upright faces, and we know that humans do not learn to processes inverted faces holistically/configurally (Robbins and McKone, 2003) even after considerably more trials than the bees were trained with. Without any evidence that the bees were performing the discrimination based on facial identity, and without an examination of the extent to which they showed an inversion effect for discriminations with other categories of objects (to test whether the face inversion effect is disproportionately large), there is no reason to suppose that bees process human faces configurally/holistically.

The other non-primates that have been tested for the inversion effect are sheep (Kendrick et al., 1995, 1996), dogs (Racca et al., 2010), and pigeons (Phelps and Roberts, 1994 – although they were tested with primate, not pigeon, faces). Phelps and Roberts trained pigeons to discriminate between pairs of faces (one of which was arbitrarily designated correct) that were either both upright or both inverted, and found that the pigeons discriminated upright and inverted human, “monkey” and “great ape” faces equally well – they showed no evidence at all of an inversion effect for these faces. A spider monkey and human participants, tested in an essentially identical fashion, showed inversion effects for all categories of primate faces, although larger effects for human faces, but this was the only category that only contained one species, so this is unsurprising (as explained later). This shows both that pigeons do not have a mechanism that responds to upright primate faces like humans do (perhaps unsurprisingly, although the authors also briefly reported a pilot failure to find an inversion effect for “bird” faces), and that upright primate faces are not intrinsically easier to discriminate than inverted primate faces (in the absence of such a mechanism).

Racca et al. studied face processing in dogs using a visual paired comparison method – a match-to-sample habituation paradigm, in which a sample stimulus is replaced by two spatially separated images, one of which matches the sample. Typically, subjects spend more time looking at the new stimulus, although in this study dogs did this for common objects and human faces, but for dog faces they actually looked more at image of the previously presented individual. Differential looking (albeit in different directions for human and dog faces) was taken as evidence of individuation. Although the matching faces are reported to be different photos of a particular individual (from slightly different perspectives), and they were manipulated to prevent matching based on brightness or contrast, from the example figures they look remarkably similar, so it is not completely convincing that the dogs were recognizing *individuals*. When the sample stimulus was upright but the test images were inverted (it is usual in human studies to have *both* sample and test stimuli upright *or* inverted, since then neither task requires an image transformation) then no significant differences in looking time for any of the stimuli was found, and so while there is evidence of a version of the inversion effect there is no evidence of a *disproportionate* inversion effect for faces (of either dogs or humans), and so no evidence of configural/holistic processing of faces by dogs.

Perhaps the best-known non-primate face research is that of Kendrick and colleagues (Kendrick et al., 1995, 1996; Peirce et al., 2000, 2001). In the original study (Kendrick et al., 1995) sheep were trained in a Y maze to choose *between* the face a of sheep and that of a human, or a sheep and a dog, etc. (with sheep choices reinforced), and so it is actually surprising that the reported inversion effect occurred at all, given that there is no need to base this discrimination on facial identity or even a configuration of features – a wide range of individual features would serve to reliably pick the sheep “face.” There is some evidence of a speed-accuracy tradeoff when the faces are inverted, and so perhaps the greater speed with which responses to inverted stimuli were made affected accuracy. Kendrick et al. (1996) improved on this methodology by training sheep to discriminate between the photos of different individual sheep (but still only single exemplars of a particular individual) with both faces upright or both faces inverted. There were no transfer tests – all pairs of stimuli were differentially reinforced on every trial, and trained in (two) different orders – and so the inversion effect reported is a consequence of being better able to *learn* to respond differentially to upright or inverted faces (the same faces were used in the upright and inverted conditions). For own-breed (but unfamiliar) sheep faces (Clun Forest), the sheep performed better when the faces were upright (approximately 85% compared to 75% performance), but with other (and unfamiliar) breed faces (Dalesbred) there was no effect of inversion (if anything it is in the wrong direction). With upright faces the sheep also found it much easier to associate rewards with particular familiar sheep than with particular unfamiliar sheep of their own-breed. This difference was not tested with inverted faces, and so, unfortunately, it is impossible to tell whether the lack of inversion effect for other breed faces is due to them being *less* like the faces with which they are familiar, or to the fact that Dalesbred sheep have many more non-configural features to distinguish individuals – including distinctive white spots on their faces and long, differentially curling horns (Clun Forest sheep have uniformly black faces and no horns). There was also no effect of inversion for a picture of an empty vs. a full food bucket, but since the presence or absence of food is signaled by a very obvious featural cue (the food), this does not really provide convincing evidence for a *disproportionate* inversion effect for sheep faces.

Parr (2011) reviews evidence for inversion effects in non-human primates (among other markers of holistic processing discussed later), and concludes that the evidence is good for common Chimpanzees, but mixed for monkeys – the other apes and prosimians have never been tested. While it is true that evidence from monkeys is mixed, *failure* to find evidence for an inversion effect can occur for many reasons other than the species in question failing to process faces holistically/configurally, and so a brief examination of the strength of the evidence is warranted.

Two of the earliest studies to attempt to reconcile previously contradictory reports of inversion effects in monkeys are those of Phelps and Roberts (1994) and Wright and Roberts (1996). Using match-to-sample and discrimination learning procedures, both studies reported inversion effects for human, ape, and monkey faces, but not with outdoor scenes, and bigger inversion effects for human faces. Phelps and Roberts used squirrel monkeys, a new world species, and Wright and Roberts used Rhesus macaques, an old-world species, suggesting a phylogenetically widespread disproportionate inversion effect for faces in monkeys, and so possibly a relatively early evolutionary origin of configural processing of faces in the primates. It is difficult to draw definitive conclusions about the relative size of the inversion effect for monkey, ape, and humans faces since, as described earlier when pigeons were the participants, the human face discriminations involved pictures of different human faces, but the “ape” and “monkey” tasks involved discriminations between different *species* that happened to be apes or monkeys. Given this, the surprising aspect of these results is that there was an inversion effect at all for ape and monkey faces (indeed there only just was in Wright and Roberts), not that it was bigger for human faces. For this reason it is perhaps premature to speculate on whether some kinds of primate *faces* might better support configural/holistic processing, as do these authors and Parr (2011).

Subsequent studies have avoided the stimulus problems of these studies, but have not yet managed to produce a clear picture of the distribution of inversion effects in primates, largely because of methodological concerns. In studies run by Parr and colleagues, for example, it seems to be true that rhesus macaques require many more training trials to acquire the match-to-sample task than do chimpanzees, which raises questions about whether they are processing the stimuli in the same way. To take an example, Parr et al. (1998) found that Chimpanzees showed an inversion effect for human and chimpanzee faces (those with which they had experience), but not for the faces of capuchin monkeys (a new world species) or cars, but that Rhesus macaques (Parr et al., 1999) tested in much the same way (but requiring *many* more trials – about 2000 per category) showed inversion effects for the rhesus faces, capuchin faces, cars, and human faces (not significant, but as large as the other primate inversion effects), but not for abstract symbols. The monkeys had had prior training on similar *tasks* but not with socially relevant stimuli, in which they may have learned to process visually presented stimuli in such tasks in a way that interfered with subsequent face discriminations. In any case, so many training trials suggests an unusual method of learning the match-to-sample procedure. All monkeys were unfortunately also tested with the same order of stimulus conditions (rhesus, shapes, capuchin, then human), and were not tested with novel exemplars, and so it is impossible to tell what role was played by particular exemplars and/or the order of testing.

Further muddying the picture of inversion effects in non-human primates is the fact that Parr et al. (2008) *did* find evidence of a disproportionate inversion effect for faces with rhesus monkeys (for human, chimp, and rhesus faces, but not for houses or clip art objects) and that when examined in detail, the chimpanzee inversion effect is somewhat odd. Parr and Heintz (2006) tested match-to-sample performance for chimpanzee faces and houses (with what appears, from the example figures, to be an unfortunately heterogeneous set of houses) with stimuli rotated in the picture plane 0, 45, 90, 135, and 180°. Although there is evidence of a substantial inversion effect for the chimp faces but not the houses at 180°, this is tempered by the fact that performance with houses was as bad as it was for inverted chimp faces at 45 and 135° – whenever the houses were at oblique angles – suggesting an unusual method of matching these stimuli. Chimp face matching was also very poor at 90° – worse than at 135° – which is not the pattern found when humans are tested with such rotations, in which there is a linear falloff in performance as orientation deviates from upright (Collishaw and Hole, 2002).

That methodological rather than species differences underlie the inconsistent results reported previously is supported by three relatively recent clear demonstrations of disproportionate inversion effects for faces in monkeys, each of which also shows evidence of holistic/configural processing being tuned to faces with which the monkeys have experience. Using a visual paired comparisons methodology (the habituation equivalent of match-to-sample), Gothard et al. (2009) using rhesus macaques (old world), and Neiworth et al. (2007), using cotton-top tamarins (a new world species), both showed clear evidence of an inversion effect for the faces of conspecifics (this was also true for human participants in Neiworth et al.) that was bigger than it was for other categories. In Gothard et al. the inversion effect for rhesus faces was bigger than for human faces, with which the monkeys had much less experience, and in Neiworth et al. the tamarins showed inversions effects for tamarin and human faces (with which they had experience), but not chimpanzee faces or objects. Similarly, using a match-to-sample procedure, but testing with novel exemplars, and so ruling out face-particular featural matching, Pokorny et al. (2011), using capuchin monkeys, found an inversion effect for capuchin and human faces (bigger for capuchins, although overall performance was also much poorer for human faces, so this could be a floor effect), but no significant inversion effect for chimpanzee faces or cars.

In summary, the most convincing evidence for a disproportionate inversion effect for faces actually comes from studies with monkeys, and there is some evidence that this effect extends to other primate faces with which the monkeys have experience. While the evidence for a disproportionate inversion effect in other species is not compelling, this may reflect differential research effort rather than genuine phylogenetic discontinuities.

## Phylogenetic Distribution – Composite Effect

The other main index of configural/holistic processing that has been tested in non-humans is the composite effect – better recognition of one half of a face (typically the top) when it is presented misaligned with the bottom half of a different individual’s face than when it is presented in alignment with a different bottom half. When the mismatching halves are presented aligned, the tendency to process them holistically interferes with our ability to extract the identity of just the top half. To our knowledge only four studies have looked for the existence of the composite effect in non-humans, and, despite some conclusions to the contrary, in only one case is the evidence compelling. The first study to test for a composite effect was run by Parr et al. (2006), using chimpanzee subjects, and although the composite stimuli were produced in the usual way, the test procedure did not actually examine the chimpanzees’ ability to recognize half-faces aligned vs. misaligned, and so did not actually test for the composite effect. Instead, in a match-to-sample procedure, aligned or misaligned chimp and human half-faces appeared as samples, and then chimps had a choice between the whole face from which the bottom half had come and the whole face from which the top half had come. Since there was no correct answer (they could match based on tops or bottoms of faces) such trials were non-differentially reinforced, a procedure that unfortunately provided no means of measuring the existence or otherwise of the composite effect for chimpanzees.

In two later studies by Taubert and Parr (2009) and Taubert (2010), Spider monkeys (a new world species) and rhesus macaques were trained in an improved match-to-sample procedure in which they learned to respond to matches in just the top half of a face (Taubert and Burke, 2008), and so could be properly tested for the composite effect – albeit a slightly unusual composite effect in that whole faces (one of which was the individual from the top half of the sample) were matched with aligned or misaligned samples. While these studies utilized an improved training technique, and incorporated testing with novel exemplars, the nature of the results renders them inconclusive. Across the two studies, spider monkeys are reported to show a composite effect for human and spider monkey faces, but not for chimp, gorilla, or sheep faces or stick objects, and rhesus macaques to *only* show a composite effect for *chimpanzee* faces (not for human, gorilla, or sheep faces or stick objects). The rhesus results seem particularly puzzling (although they were not actually tested with rhesus faces), but in fact, there is no reason to suppose that any of the results reflect the standard composite effect, since in each case where a significant composite effect is reported it is caused by substantially *below* chance performance on aligned trials. For both species of monkey judging monkey half-faces, this is as low as 25% – as far from chance as the *best* misaligned performance (75%) – a result for which there is no straightforward explanation. An additional puzzle is that *without any further training* one of the spider monkeys studied by Taubert managed to perform above chance (with just 24 trials in each category) when the composite stimuli were inverted, regardless of whether the to-be-matched half was that with the eyes (as it had been for every training trial, but which were now at the bottom of the image) or with the chin. Taubert describes this as “remarkable,” but given the trouble other monkeys have been reported to have with match-to-sample tasks (Parr, 2011), the significantly below chance performance reported for these individuals in the immediately preceding experiment (and with no further training), and the *inevitability* of errors whilst acquiring feedback about the new contingency (with no way for the monkey to even know whether an inverted stimulus was from a chin-match or an eye-match trial), “remarkable” is perhaps an understatement. The result, like the 25% performance on aligned trials in experiment 1, is, if not inexplicable, then at least unexplained. Whatever accounts for the curious results in these experiments, it is clear that they do not provide convincing evidence of a composite effect for the monkeys tested.

Despite attempts to dismiss the finding because the dependent variable used was eye movements (Taubert, 2010; Parr, 2011), the only good evidence for the composite effect in non-humans actually comes from a study by Dahl et al. (2007) using Rhesus macaques. Using a slightly modified dishabituation or rebound from adaptation paradigm as an indirect index of recognition (but with the advantage of requiring no training to recognize individuals or learn a match-to-sample task), monkeys adapted to either an aligned or misaligned picture of an unknown conspecific, and were then shown the same top half aligned (if it had been aligned at adaptation) or misaligned (if it had been misaligned) paired with a new bottom half. Monkeys showed greater rebound from adaptation (dishabituation) for aligned than for misaligned composites, suggesting the perception of a new individual in the aligned condition. Although marmoset faces were used in an earlier experiment in this study, to test for untrained, entry-level classification of faces, only rhesus faces were used in the composite task, and so we have no data on the extent to which this holistic processing extends to the faces of other species.

## Holistic Processing in Non-Humans

Much of this review has focused on the fact that there are frequently methodological reasons for questioning the conclusiveness of the failures (or successes) to demonstrate holistic/configural processing of faces in many of the species that have been tested. In addition to the limitations already discussed, whenever a photograph is used with non-humans (which is how all such experiments have been run) we need to wonder about whether the subject actually sees it as a face, and bases its discrimination performance on this aspect. Humans have a lifetime of experience with such stimuli, and so have learned to effortlessly see the image as a depiction of a real object. In contrast, if the first exposure an animal has to a photograph is in a discrimination experiment, in the absence an explicit attempt to teach them that the images depict objects, by reinforcing selection of the same *individual* (or object) depicted in multiple different photographs, for example, we cannot rule out the possibility that they are just responding to particular *features* in the images, and so cannot properly interpret failures to find inversion effects as an absence of holistic/configural processing of *faces*.

Even with these methodological considerations in mind, there is quite good evidence of a disproportionate inversion effect for faces in two new world monkey species (cotton-top tamarins and capuchin monkeys), an old-world monkey (rhesus macaques), and of course, in humans, but no conclusive evidence yet of such an effect for a non-primate. Given that the lineages of new world monkeys and old-world monkeys (and apes, including humans) diverged about 40 million years ago, this either suggests an old adaptation for the kind of face processing that underpins the disproportionate inversion effect (which *may* not even be unique to primates), or something about the social ecology of humans, rhesus macaques, cotton-top tamarins, and capuchins that selected for this kind of face processing. It is not immediately obvious what this social ecological commonality might be (humans and rhesus live in much bigger groups, for example), and so perhaps the former alternative is more likely, but without more thorough, methodologically sound attempts to test related species we cannot answer this question definitively. Of the other species tested to date, we do not have definitive evidence that they do *not* show a disproportionate inversion effect, only that there is insufficient evidence to conclude that they *do*. The most straightforward way to test between a more modest version of the idea that such processing is phylogenetically old or is due to different social ecologies would be to compare the disproportionate inversion effect in the extant apes, which have dramatically different social structures, ranging from essentially solitary orang-utans, to small familial bands in gorillas and gibbons, and gregarious, fission-fusion groupings in common chimpanzees and bonobos.

The only two species for which we have strong evidence of a composite effect are rhesus macaques and humans, each for the faces of their own species. This is an obvious area where again, it would be useful to measure the composite effect (ideally for the faces of a range of species) in at least the extant apes, and possibly some new world monkeys to examine the phylogenetic extent of the effect. It would also be worthwhile looking for evidence of this effect in the face processing of other social species that recognize each other using face-based visual information. At the moment, for understandable reasons, comparative efforts are characterized by convenience sampling – testing species that researchers happen have easy access to – but a more theoretically driven selection of test species will be necessary if we are to understand the selective forces that have shaped face-sensitive mechanisms.

A complementary approach to the question of the phylogenetic distribution of the holistic processing of faces, which blends into the next section considering evidence that the face-processing mechanism in humans has adaptation-signifying properties, is to examine the kinds of face to which holistic/configural processing in humans extends. Burke et al. (2012) explored the size of the inversion effect and the composite effect using three different successive same-different tasks (matching top halves in the composite task) for a range of different face stimuli. In the first experiment, the inversion effect was large and robust (in errors, reaction time, and efficiency measures) for the faces of humans and chimpanzees, slightly smaller for the faces of marmosets, significantly smaller again for the faces of gibbons and cats and for homogenous plants (all small agaves), and absent for images of apples. In experiment 2, probing the effect with primate faces more fully, there were again very large inversion effects for the faces of humans, common chimpanzees, and marmosets, smaller effects for the faces of bonobos and gibbons, and almost no effect for the faces of gorillas and orang-utans. This study shows a disproportionate inversion effect for human faces that extends to the faces of common chimpanzees, and to a lesser extent to the faces of a range of other primates, but *not* to those of gorillas or orang-utans. This does not map neatly onto phylogeny, nor familiarity, given that marmosets are likely seen much less frequently than gorillas, orang-utans, or even bonobos – although common chimpanzees are probably the most frequently encountered non-human primate, and so perhaps there is some role for familiarity. This suggests that the mechanism that is responsible for producing the disproportionate inversion effect for faces in humans is tuned equally well (at least in our sample) to human and common chimpanzee faces, and to a lesser extent to the faces of marmosets, gibbons, and bonobos. It is unknown to what extent this tuning is shaped by experience.

In contrast, the composite effect (tested with human, bonobo, common chimp, gorilla, orang-utans, gibbon, and marmoset faces) was *specific* to the faces of humans – there was *no* evidence of a composite effect for the face of *any* other species, and in fact it was slightly negative for marmoset faces, which had shown a large inversion effect. This is a clear dissociation between the mechanism that produces the disproportionate inversion effect and the one that produces the composite effect, in that, at least in the humans we tested, the composite effect is exclusive to *human faces* (not just to *faces*, as has been suggested previously – Robbins and McKone, 2007). Given that there is quite good evidence that rhesus macaques also show the composite effect (only tested with faces of their own species), we have evidence that the holistic processing that produces the composite effect is present in two old-world primates (albeit one a monkey and one an ape), and that it may be restricted to own-species faces. As suggested earlier, more species need to be tested more carefully and with more faces to more fully understand the nature of this kind of holistic processing even in the primates.

## Signs of Special Design

Testing whether human face processing shows special design features is difficult because although there is a widespread (largely implicit) assumption that the underlying mechanism is *for identity* recognition, the putative neural substrate of such processing (the fusiform face area), and the behavioral markers of holistic processing (like the inversion and composite effects) have not been tested thoroughly enough with other possibilities to be confident that this is true. Both comparative studies and studies focused on revealing signs of special design are hampered by the fact that we do not actually have a very clear idea of the *function* of holistic/configural processing, and so it is difficult to make predictions about what species ought to show it, or for what *kinds* of judgments it is necessary.

While holistic/configural processing was first discovered in the context of extracting *identity* information this is not necessarily the source of face-based information for which such processing is most obviously useful, or for which it evolved. It is true that faces have broadly the same features in the same first order configuration and differ primarily in terms of the second order configuration of those features (Maurer et al., 2002), but it is also true that some of the features themselves serve as quite reliable identification cues (Collishaw and Hole, 2000). In fact, given that identity *could* be signaled by a range of obvious featural cues (and *is* in some species – Tibbetts and Dale, 2007), it is worth asking why holistic/configural information is used when identifying individuals, and why individuals do not signal their identity more obviously. One unexplored possibility is that there might be costs as well as benefits to signaling identity (Tibbetts and Dale, 2007), as there are with all evolved signals, and so perhaps the signaling of identity-based on configural cues represents a compromise between these. It could be, for example, that signaling identity to strangers is costly, but to in-group members is beneficial, in which case we would expect a system of identity signaling to evolve that reflected this tradeoff. Given that both recognition performance and holistic processing increase with experience with particular kinds of faces (e.g., Michel et al., 2006), such a tradeoff could explain the evolution of a holistic signaling of identity.

One of the few papers to explicitly consider the evolutionary origin of holistic processing of facial identity was that of McKone (2009). Based on evidence that holistic processing of faces for identity is most evident at simulated distances of between 2 and 10 m, McKone reasonably proposed that this is the distance at which identification is important to one’s safety, arguing that subtle expression differences (as an example of another source of face-based socially important information) would be more detectable at conversational distances of around 1 m. While this logic is sound, and may well explain the range over which *identity*-based holistic processing operates, and is exactly the kind of evidence needed to reveal “special design features” of holistic identity processing, we know that *expression* information is also extracted holistically, since such judgments show a top-bottom composite effect (Calder et al., 2000). Indeed, perceiving many expressions (particularly complex non-“standard” ones) probably *requires* integration across the whole face, and some expressions show differential patterns of lateralized production depending on whether they are genuine or faked (Indersmitten and Gur, 2003), and so left-right integration of expression information might also be important for detecting dishonest social partners, an ability with obvious potential fitness consequences.

Not only is there good evidence that expression processing is holistic/configural (Calder et al., 2000), there is also evidence from single cell recording studies with monkeys of some expression sensitivity in temporal lobe “identity” sensitive regions of the brain (Perrett et al., 1992; Sugase et al., 1999), brain-damage-based identity processing difficulties (prosopagnosia) frequently co-occur with expression processing difficulties (Calder and Young, 2005), and expression aftereffects are partially tuned to facial identity, suggesting overlapping neural processing (Campbell and Burke, 2009). This suggests that both the specialized neural area and the behavioral markers of “special” processing are not exclusively related to extracting identity information, which, in turn, raises questions about what holistic/configural processing might then be *for*.

A possibility that has not been considered previously (in part because the evolutionary origin of holistic processing has not been much considered) is that the fact that identity is processed holistically might actually be a side-effect of selection for other mechanisms that need to extract other kinds of face-based information holistically – or this could have been how holistic processing started for faces, and then identity processing co-opted the existing mechanism(s). Along these lines, Robbins and Coltheart (2012) suggested that integrating information across the face and across the body, producing holistic processing, seems likely to be important for a range of communicative functions, and for judging symmetry (known to be important in mate choice). Below we list six potential candidates, other than identity, for the evolutionary origin of the holistic processing of faces, since each of them requires some degree of integration of information from different regions of the face, but there are likely others.

(i) Expression (as already discussed) and other face-based signals of behavioral intent, perhaps including assessments of honesty, trustworthiness, approachability/friendliness, dangerousness, aggressiveness, etc. (Robbins and Coltheart, 2012).
(ii) Sex-judgments, since sexually dimorphic signals relate to the *relative* size of jaws and upper faces (Burke and Sulikowski, 2010).
(iii) Masculinity and femininity of faces, which signal sex-hormone levels, and so are important in mate-choice decisions, and therefore attractiveness judgments (Burke and Sulikowski, 2010).
(iv) Facial symmetry, another important determinant of attractiveness (Thornhill and Gangestad, 1994), and which obviously involves integrating information from the left and right halves of a face (as suggested by Robbins and Coltheart, 2012).
(v) Relatedness (DeBruine, 2005), biologically important as a basis of kin-selection (and incest avoidance), and which also likely depends on integrating information across the whole face.
(vi) Age, since it is primarily the configuration of the features of a face and the face outline that shift during development (Pittenger et al., 1979).

Each of these is a source of face-based information with obvious survival and reproductive import, and which depends on integrating information across the whole face. Any, or all, of them could have provided the original selection pressure for processing faces holistically. In order to discover which are most important, and to develop and test theories of the evolution of this mechanism, we will need to run experiments examining the role of holistic/configural processing in extracting each of these sources of face-based information, across a range of carefully selected (and well tested) species. To date, almost all of the research has been conducted on one species (humans) extracting one source of face-based information (identity).

A few studies have used the composite effect to examine the role of holistic processing in general attractiveness judgments (Abbas and Duchaine, 2008), judgments of sex (Baudouin and Humphreys, 2006), of Age (Hole and George, 2011), and of trustworthiness (Todorov et al., 2010), and each concludes that such information is extracted holistically, but because they are isolated studies, using idiosyncratic methodologies, the conclusions are difficult to evaluate. For example, Todorov et al. used a statistically derived, computer-generated manipulation of “trustworthiness,” in which trustworthiness co-varies with expression, and so perhaps the composite effect they measured is operating at the level of expression perception rather than trustworthiness perception itself. Similarly, the age composite task of Hole and George contained no misaligned condition, and so general interference from the to-be-ignored half (in the aligned condition) may account for the effect they reported, and the attractiveness study of Abbas and Duchaine did contain a “misaligned” condition, but rather than laterally offsetting the two halves, they were presented vertically aligned, but with the top of the face below the bottom, making it difficult to compare their results to those of identity composite effect studies. Baudouin and Humphreys used a conventional composite task paradigm, but 15 of their 16 participants were female (in a sex-judgment task), and so it is possible that the results may not generalize to males.

## Conclusion

Attempting to derive an understanding of the evolution of holistic/configural processing of faces using the evidence so far gathered is challenging for two main reasons. First, many psychology and neuroscience researchers are naturally focused on proximate rather than ultimate explanations, and so have examined *how* face processing works, rather than *why* it works the way it does. This focus has led to a widespread misapprehension that evidence supporting an experience-independent face-specific neural area for face processing is necessary to conclude that the holistic processing of faces is an evolved adaptation. Whichever side of this debate particular researchers fall, each seems satisfied that adopting one position or the other is equivalent to deciding that our ability to process faces is either an evolved ability *or* a learned ability. We have argued, in common with modern perspectives on the evolution of psychological/neural mechanisms (e.g., McNamara and Houston, 2009; Barrett, 2012), that this is not a reliable way of assessing whether a mechanism is an evolved adaptation, and that instead, what is needed is a careful analysis of the costs and benefits of the mechanisms within a known social/environmental context, followed by tests of the extent to which the mechanism is distributed in the hypothesized way across different species with known relatedness and relevant ecologies (in this case social ecology). The second challenge is that although some of the comparative studies have been designed to test the phylogenetic and/or ecological distribution of holistic face processing, none has done this with a clear idea of the *function* of the face-processing mechanisms they are examining, being instead motivated to examine whether holistic face processing is unique to humans. This is compounded by methodological shortcomings calling into question many of the conclusions drawn.

With these challenges in mind, the broad conclusions of the current review are that there is good evidence of an inversion effect in identity judgments in humans and old world and new world monkeys (that may be biggest for faces with which the test species is familiar), but no genuinely convincing evidence yet of such an effect in a non-primate or even another ape species, and good evidence of a composite effect in identity judgments in rhesus macaques (an old-world monkey) and humans. In humans, Burke et al. (2012) found that the inversion effect in identity judgments is as strong for the faces of common chimpanzees as it is for the faces of humans, almost as large for the faces of marmosets (a distantly related new world species), and smaller or absent for other ape species. The composite effect, in contrast, is *exclusive* to human faces, suggesting that the kind of processing that underpins the composite effect in humans operates on a very restricted input (only human faces), a sign of “special design,” consistent with the underlying mechanism being an evolved adaptation. It would be interesting to know the extent to which this own-species specificity applies to other species that demonstrate a reliable composite effect (so far only rhesus macaques). Currently, the data do not allow us to draw any firm conclusions even about which species show the inversion effect and composite effect, let alone trying to map out when in the human lineage the mechanisms underpinning such processing evolved. This is, however, an achievable goal, if related species (initially of apes) are carefully tested with sound methodologies and high-quality stimuli.

A complimentary approach to trying to understand the evolution of a particular mechanism is to attempt to measure the costs and benefits of the operation of the mechanism, in order to understand the constraints on selection that shaped its evolution. Usually this is done with a clear idea of the functional benefit of the mechanism in mind, but as we have argued, it is currently impossible to be sure even which *kind* of face-processing benefits most from holistic processing, and so a cost-benefit analysis is premature. At the moment we have good evidence from the composite task that identity-, expression- (at least the six “basic” expressions), and sex-judgments (at least by females) involve holistic processing, and some evidence that this might also be true for judgments of age, attractiveness, and trustworthiness. The mere fact that holistic processing extends beyond identity recognition raises the possibility it initially evolved to extract a different kind of face-based information, and the fact that face-recognition ability shows substantial heritability raises the additional possibility that it may have been sexual selection (serving mate-choice or intra-sexual competition functions) rather than natural selection (serving survival or direct reproductive functions) that led to the evolution of holistic face processing. If sexual selection is responsible for the evolution of holistic processing, then we might expect sex differences in holistic processing, especially for some kinds of judgments.

To properly address the evolution of holistic face processing, and to consequently develop a proper understanding of its *function*, it might be instructive to forget that it was first uncovered using identity judgments, and to re-examine the question from a functional perspective – to ask ourselves what kind of face-based information is most likely to benefit from integrating information across the face? From this perspective, identity seems a much less likely candidate than subtle expression (or communicative functions in general), in which a multitude of nuanced meanings can be conveyed by different combinations of dynamically unfolding configurations of facial distortions. Judgments of subtle variations in masculinity and femininity, which are known to be important in mate-choice/attractiveness decisions, also seem more likely to *require* integration of information across the face than judgments of identity, a possibility consistent with evidence that holistic processing may be at least partly a consequence of sexual selection. These are simply speculations based on a consideration of the *possible* function of holistic integration of information across the face. This kind of speculation is useful, but to become testable hypotheses about the evolution of holistic processing they need to be informed and constrained by good data about which precise kinds of face perception involve holistic processing (manipulations of the facial factors known to affect attractiveness, for example, rather than just overall attractiveness), and the species that show such effects.

## Conflict of Interest Statement

The authors declare that the research was conducted in the absence of any commercial or financial relationships that could be construed as a potential conflict of interest.

## References

[B1] Abbas Z.-A. Duchaine B. (2008). The role of holistic processing in judgments of facial attractiveness. Perception 37, 1187–1196 10.1068/p5984 18853555

[B2] Arnold S. J. (1983). “Sexual selection: the interface of theory and empiricism,” in Mate Choice, ed. Bateson P. P. G. (Cambridge: Cambridge University Press), 67–107

[B3] Barrett H. C. (2012). A hierarchical model of the evolution of human brain specializations. Proc. Natl. Acad. Sci. U.S.A. 109, 10733–10740 10.1073/pnas.1208417109 22723350 PMC3386868

[B4] Baudouin J.-Y. Humphreys G. W. (2006). Configural information in gender categorisation. Perception 35, 531–540 10.1068/p3403 16700294

[B5] Breed M. D. (1998). Recognition pheromones on the honey bee. Bioscience 48, 463–470 10.2307/1313230

[B6] Burke D. Everingham P. Rogers T. Hinton M. Hall-Aspland S. (2001). Perceptual grouping in two visually reliant species; Humans (*Homo sapiens*) and Australian sea lions (*Neophoca cinerea*). Perception 30, 1093–1106 10.1068/p3239 11694085

[B7] Burke D. Sulikowski D. (2010). A new viewpoint on the evolution of sexually dimorphic human faces. Evol. Psychol. 8, 573–585 22947821 10.1177/147470491000800404 PMC10426831

[B8] Burke D. Sulikowski D. Willis M. McKone E. Favelle S. (2012). Human faces are special for humans. Paper Presented at the 24th Annual Meeting of the Human Behavior and Evolution Society Albuquerque, NM

[B9] Calder A. J. Young A. W. (2005). Understanding the recognition of facial identity and facial expression. Nat. Rev. Neurosci. 6, 641–651 10.1038/nrn1724 16062171

[B10] Calder A. J. Young A. W. Keane J. Dean M. (2000). Configural information in facial expression perception. J. Exp. Psychol. Hum. Percept. Perform. 26, 527–551 10.1037/0096-1523.26.2.527 10811161

[B11] Campbell J. Burke D. (2009). Evidence that identity-dependent and identity-independent neural populations are recruited in the perception of five basic emotional facial expressions. Vision Res. 49, 1532–1540 10.1016/j.visres.2009.03.009 19303422

[B12] Collishaw S. M. Hole G. J. (2000). Featural and configurational processes in the recognition of faces of different familiarity. Perception 29, 893–909 10.1068/p2949 11145082

[B13] Collishaw S. M. Hole G. J. (2002). Is there a linear or a nonlinear relationship between rotation and configural processing of faces? Perception 31, 287–296 10.1068/p3195 11954691

[B14] Coulon M. Baudoin C. Heyman Y. Deputte B. L. (2011). Cattle discriminate between familiar and unfamiliar conspecifics by using only head visual cues. Anim. Cogn. 14, 279–290 10.1007/s10071-010-0361-6 21132446

[B15] Dahl C. D. Logothetis N. K. Hoffman K. L. (2007). Individuation and holistic processing of faces in rhesus monkeys. Proc. R. Soc. Lond. B Biol. Sci. 274, 2069–2076 10.1098/rspb.2007.0477 PMC1919404 17609192

[B16] DeBruine L. (2005). Trustworthy but not lust-worthy: context-specific effects of facial resemblance. Proc. R. Soc. Lond. B Biol. Sci. 272, 919–922 10.1098/rspb.2004.3003 PMC1564091 16024346

[B17] Dyer A. G. Neumeyer C. Chittka L. (2005). Honeybee (*Apis mellifera*) vision can discriminate between and recognise images of human faces. J. Exp. Biol. 208, 4709–4714 10.1242/jeb.01929 16326952

[B18] Gauthier I. Bukach C. (2007). Should we reject the expertise hypothesis? Cognition 103, 322–330 10.1016/j.cognition.2006.03.002 16780825

[B19] Gauthier I. Tarr M. J. (1997). Becoming a ‘greeble’ expert: exploring mechanisms for face recognition. Vision Res. 37, 1673–1682 10.1016/S0042-6989(96)00286-6 9231232

[B20] Gothard K. M. Brooks K. N. Peterson M. A. (2009). Multiple perceptual strategies used by macaque monkeys for face recognition. Anim. Cogn. 12, 155–167 10.1007/s10071-008-0179-7 18787848

[B21] Hole G. George P. (2011). Evidence for holistic processing of facial age. Vis. Cogn. 19, 585–615 10.1080/13506285.2011.562076

[B22] Indersmitten T. Gur R. C. (2003). Emotion processing in chimeric faces: hemispheric asymmetries in expression and recognition of emotions. J. Neurosci. 23, 3820–3825 12736352 10.1523/JNEUROSCI.23-09-03820.2003 PMC6742199

[B23] Kendrick K. M. Atkins K. Hinton M. R. Broad K. D. Fabre-Nys C. Keverne B. (1995). Facial and vocal discrimination in sheep. Anim. Behav. 49, 1665–1676 10.1016/0003-3472(95)90088-8

[B24] Kendrick K. M. Atkins K. Hinton M. R. Heavens P. Keverne B. (1996). Are faces special for sheep – evidence from facial and object discrimination-learning tests showing effects of inversion and social familiarity. Behav. Processes 38, 19–35 10.1016/0376-6357(96)00006-X 24897627

[B25] Leopold D. A. Rhodes G. (2010). A comparative view of face perception. J. Comp. Psychol. 124, 233–251 10.1037/a0019460 20695655 PMC2998394

[B26] Maurer D. Grand R. L. Mondloch C. J. (2002). The many faces of configural processing. Trends Cogn. Sci. (Regul. Ed.) 6, 255–260 10.1016/S1364-6613(02)01903-4 12039607

[B27] McKone E. (2009). Holistic processing for faces operates over a wide range of sizes but is strongest at identification rather than conversational distances. Vision Res. 49, 268–283 10.1016/j.visres.2008.10.020 19022276

[B28] McKone E. Kanwisher K. Duchaine B. (2007). Can generic expertise explain special processing for faces? Trends Cogn. Sci. 11, 8–15 10.1016/j.tics.2006.11.002 17129746

[B29] McKone E. Palermo R. (2010). A strong role for nature in face recognition. Proc. Natl. Acad. Sci. U.S.A. 107, 4795–4796 10.1073/pnas.1000567107 20212105 PMC2841894

[B30] McKone E. Robbins R. (2007). The evidence rejects the expertise hypothesis: reply to Gauthier & Bukach. Cognition 103, 331–336 10.1016/j.cognition.2006.05.014 16842769

[B31] McNamara J. M. Houston A. I. (2009). Integrating function and mechanism. Trends Ecol. Evol. 24, 670–675 10.1016/j.tree.2009.05.011 19683827

[B32] Michel C. Rossion B. Han J. Chung C. Caldara R. (2006). Holistic processing is finely tuned for faces of one’s own race. Psychol. Sci. 17, 608–615 10.1111/j.1467-9280.2006.01752.x 16866747

[B33] Neiworth J. J. Hassett J. M. Sylvester C. J. (2007). Face processing in humans and new world monkeys: the influence of experiential and ecological factors. Anim. Cogn. 10, 125–134 10.1007/s10071-006-0045-4 16909230

[B34] Parr L. A. (2011). The evolution of face processing in primates. Philos. Trans. R. Soc. Lond. B Biol. Sci. 366, 1764–1777 10.1098/rstb.2010.0358 21536559 PMC3130377

[B35] Parr L. A. Dove T. Hopkins W. D. (1998). Why faces may be special: evidence for the inversion effect in chimpanzees (*Pan troglodytes*). J. Cogn. Neurosci. 10, 615–622 10.1162/089892998563013 9802994

[B36] Parr L. A. Heintz M. (2006). The perception of unfamiliar faces and houses by chimpanzees: influence of rotation angle. Perception 35, 1473–1483 10.1068/p5455 17286118

[B37] Parr L. A. Heintz M. Akamagwuna U. (2006). Three studies of configural face processing by chimpanzees. Brain Cogn. 62, 30–42 10.1016/j.bandc.2006.03.006 16678323 PMC2826113

[B38] Parr L. A. Heintz M. Pradhan G. (2008). Rhesus monkeys (*Macaca mulatta*) lack face expertise. J. Comp. Psychol. 122, 390–402 10.1037/0735-7036.122.4.390 19014263 PMC2859434

[B39] Parr L. A. Winslow J. T. Hopkins W. D. (1999). Is the inversion effect in rhesus monkeys face specific? Anim. Cogn. 2, 123–129 10.1007/s100710050032

[B40] Peirce J. W. Leigh A. E. Kendrick K. M. (2000). Configurational coding, familiarity and the right hemisphere advantage for face recognition in sheep. Neuropsychologia 38, 475–483 10.1016/S0028-3932(99)00088-3 10683397

[B41] Peirce J. W. Leigh A. E. daCosta A. P. C. Kendrick K. M. (2001). Human face recognition in sheep: lack of configurational coding and right hemisphere advantage. Behav. Processes 55, 13–26 10.1016/S0376-6357(01)00158-9 11390088

[B42] Perrett D. I. Hietanen J. K. Oram M. W. Benson P. J. (1992). Organization and functions of cells responsive to faces in the temporal cortex. Philos. Trans. R. Soc. Lond. B Biol. Sci. 335, 23–30 10.1098/rstb.1992.0003 1348133

[B43] Phelps M. T. Roberts W. A. (1994). Memory for pictures of upright and inverted faces in humans (*Homo sapiens*), squirrel monkey (*Saimiri sciureus*), and pigeons (*Columba livia*). J. Comp. Psychol. 108, 114–125 10.1037/0735-7036.108.2.114 8026162

[B44] Pittenger J. B. Shaw R. E. Mark L. S. (1979). Perceptual information for the age-level of faces as a higher-order invariant of growth. J. Exp. Psychol. Hum. Percept. Perform. 5, 478–493 10.1037/0096-1523.5.3.478 528953

[B45] Pokorny J. K. Webb C. E. de Waal F. B. M. (2011) , An inversion effect modified by expertise in capuchin monkeys. Anim. Cogn. 14, 839–846 10.1007/s10071-011-0417-2 21573949

[B46] Proops L. McComb K. Reby D. (2009). Cross-modal individual recognition in domestic horses (*Equus caballus*). Proc. Natl. Acad. Sci. U.S.A. 106, 947–951 10.1073/pnas.0809127105 19075246 PMC2630083

[B47] Racca A. Amadei E. Ligout S. Guo K. Meints K. Mills D. (2010). Discrimination of human and dog faces and inversion responses in domestic dogs (*Canis familiaris*). Anim. Cogn. 13, 525–533 10.1007/s10071-009-0303-3 20020168

[B48] Robbins R. A. Coltheart M. (2012). Left-right holistic integration of human bodies. Q. J. Exp. Psychol. (Hove) 65, 1962–1974 10.1080/17470218.2012.674145 22524623

[B49] Robbins R. McKone E. (2003). Can holistic processing be learned for inverted faces? Cognition 88, 79–107 10.1016/S0010-0277(03)00020-9 12711154

[B50] Robbins R. McKone E. (2007). No face-like processing for objects-of-expertise in three behavioural tasks. Cognition 103, 331–336 10.1016/j.cognition.2006.02.008 16616910

[B51] Ryan C. M. E. Lea S. E. G. (1994). Images of conspecifics as categories to be discriminated by pigeons and chickens: slides, video tapes, stuffed birds and live birds. Behav. Processes 33, 155–176 10.1016/0376-6357(94)90064-7 24925244

[B52] Sugase Y. Yamane S. Ueno S. Kawano K. (1999). Global and fine information coded by single neurons in the temporal visual cortex. Nature 400, 869–873 10.1038/23703 10476965

[B53] Taubert J. (2010). Evidence of human-like holistic face processing in spider monkeys (*Ateles geoffroyi*). J. Comp. Psychol. 124, 57–65 10.1037/a0017704 20175597

[B54] Taubert J. Burke D. (2008). A face recognition task: how spider monkeys (*Ateles Geoffroyi*) and humans match composite stimuli. Paper Presented at the 23rd Congress of the International Primatological Society, Edinburgh

[B55] Taubert J. Parr L. A. (2009). Visual expertise does not predict the composite effect across species: a comparison between spider (*Ateles geoffroyi*) and rhesus (*Macaca mulatta*) monkeys. Brain Cogn. 71, 187–195 10.1016/j.bandc.2009.09.002 19815323 PMC2783790

[B56] Thornhill R. Gangestad S. W. (1994). Human fluctuating asymmetry and sexual behavior. Psychol. Sci. 5, 297–302 10.1111/j.1467-9280.1994.tb00629.x

[B57] Tibbetts E. A. (2002). Visual signals of individual identity in the wasp Polistes fuscatus. Proc. R. Soc. Lond. B Biol. Sci. 269, 1423–1428 10.1098/rspb.2002.2031 PMC1691056 12137570

[B58] Tibbetts E. A. Dale J. (2007). Individual recognition: it is good to be different? Trends Ecol. Evol. 22, 529–537 10.1016/j.tree.2007.09.001 17904686

[B59] Todorov A. Loehr V. Oosterhof N. N. (2010). The obligatory nature of holistic processing of faces in social judgments. Perception 39, 514–532 10.1068/p6501 20514999

[B60] Van Dyk D. A. Evans C. S. (2007). Familiar-unfamiliar discrimination based on visual cues in the jacky dragon, Amphibolurus muricatus. Anim. Behav. 74, 33–44 10.1016/j.anbehav.2006.06.018

[B61] Watanabe S. (1993). Object-picture equivalence in the pigeon: an analysis with natural concept and pseudoconcept discrimination. Behav. Processes 30, 225–232 10.1016/0376-6357(93)90134-D 24896946

[B62] Watanabe S. Ito Y. (1991). Discrimination of individuals in pigeons. Bird Behav. 9, 20–29 10.3727/015613890791749136

[B63] Wright A. A. Roberts W. A. (1996). Monkey and human face perception: inversion effects for human faces but not for monkey faces or scenes. J. Cogn. Neurosci. 8, 278–290 10.1162/jocn.1996.8.3.278 23968152

[B64] Yin R. K. (1969). Looking at upside-down faces. J. Exp. Psychol. 81, 141–145 10.1037/h0027474

